# Exploiting the Richness of Environmental Waterborne Bacterial Species to Find Natural *Legionella pneumophila* Competitors

**DOI:** 10.3389/fmicb.2018.03360

**Published:** 2019-01-15

**Authors:** Marie-Hélène Corre, Vincent Delafont, Anasthasia Legrand, Jean-Marc Berjeaud, Julien Verdon

**Affiliations:** Laboratoire Ecologie et Biologie des Interactions, UMR CNRS 7267, Université de Poitiers, Poitiers, France

**Keywords:** *Legionella*, freshwater environments, *Pseudomonas*, bacterial community, inhibition profile, volatile organic compound, antimicrobials

## Abstract

*Legionella pneumophila* is one of the most tracked waterborne pathogens and remains an important threat to human health. Despite the use of biocides, *L. pneumophila* is able to persist in engineered water systems with the help of multispecies biofilms and phagocytic protists. For few years now, high-throughput sequencing methods have enabled a better understanding of microbial communities in freshwater environments. Those unexplored and complex communities compete for nutrients using antagonistic molecules as war weapons. Up to now, few of these molecules were characterized in regards of *L. pneumophila* sensitivity. In this context, we established, from five freshwater environments, a vast collection of culturable bacteria and investigated their ability to inhibit the growth of *L. pneumophila*. All bacterial isolates were classified within 4 phyla, namely Proteobacteria (179/273), Bacteroidetes (48/273), Firmicutes (43/273), and Actinobacteria (3/273) according to 16S rRNA coding sequences. *Aeromonas*, *Bacillus*, *Flavobacterium*, and *Pseudomonas* were the most abundant genera (154/273). Among the 273 isolates, 178 (65.2%) were shown to be active against *L. pneumophila* including 137 isolates of the four previously cited main genera. Additionally, other less represented genera depicted anti-*Legionella* activity such as *Acinetobacter*, *Kluyvera*, *Rahnella*, or *Sphingobacterium*. Furthermore, various inhibition diameters were observed among active isolates, ranging from 0.4 to 9 cm. Such variability suggests the presence of numerous and diverse natural compounds in the microenvironment of *L. pneumophila*. These molecules include both diffusible secreted compounds and volatile organic compounds, the latter being mainly produced by *Pseudomonas* strains. Altogether, this work sheds light on unexplored freshwater bacterial communities that could be relevant for the biological control of *L. pneumophila* in manmade water systems.

## Introduction

Water is essential to sustain life and granting free access to drinking-water is considered a basic human right. Therefore, water sources used for human consumption must be biologically safe, to avoid any risk for health. Indeed, the most common and widespread health risk associated with drinking water is represented by infectious diseases caused by pathogenic bacteria, viruses and parasites (World Health Organization, 2011). Most waterborne pathogens are introduced into drinking water supplies by the classic fecal-water-oral route of transmission (Ashbolt, 2015). However, other non-fecal but potentially pathogenic microorganisms like *Legionella* sp., non-tuberculous mycobacteria, *Pseudomonas aeruginosa* or free-living amoebae, may colonize engineered water systems (Falkinham et al., 2015b; Wang et al., 2017). In hot water systems and cooling towers, bacteria of the genus *Legionella* represent a particular concern and remain one of the most tracked agents, being considered as opportunistic premise plumbing pathogens (Falkinham et al., 2015a). They are part of the natural flora in many freshwater environments (i.e., rivers, streams) where they occur in relatively low numbers (Declerck et al., 2009; Declerck, 2010). *L. pneumophila* is recognized as responsible for Legionnaires’ disease (LD), a life-threatening pneumonia, and a mild form of illness named Pontiac fever (Diederen, 2008). Furthermore, the serogroup 1 is responsible for 82.9% of the LD cases in Europe (Beauté, 2017; Hamilton et al., 2018) and for over 80% of the cases worldwide (Yu et al., 2002; Newton et al., 2010). Species other than *pneumophila* account usually for less than 10% of human infections, and mostly involve *L. micdadei*, *L. bozemanii*, and *L. longbeachae* (Beauté, 2017). Some water safety plans in distribution systems, recommended by the World Health Organization, were implemented (Parr et al., 2015) such as the one published by the ASHRAE (American Society of Heating, Refrigerating, and Air-Conditioning Engineers) in 2015. This plan focuses on hazards and hazardous events for implementing control measures which aim at avoiding *Legionella* transmission and limiting its proliferatio (American Society of Heating Refrigerating and Air-Conditioning Engineers, 2015).

Persistence and colonization of *Legionella* sp. in engineered water systems is mediated by the presence of multispecies biofilms and protists like free-living amoebae (Abu Khweek and Amer, 2018). Moreover, protists serve as a natural playground for *L. pneumophila*, “training” bacteria to resist phagocytic destruction (Boamah et al., 2017). The cozy intracellular microenvironment provided by protists host cells also protects *L. pneumophila* from adverse environmental conditions representing as well a nutrient-rich replicative niche. Indeed, this intracellular lifestyle protects bacteria from being efficiently killed by water disinfection procedures (Dupuy et al., 2011). However, as mentioned by the French law n° 2009-967 (2009/08/03) adopted from discussions of the “Grenelle de l’environnement,” the control of disinfection by-products must be improved in order to avoid or limit exposure to potentially undesirable chemicals while maintaining a safe water sanitation process. In line with this aim, the description of new natural antibacterial agents active against *L. pneumophila* is a promising strategy.

To date, few studies have shown that various natural compounds efficiently inhibit *L. pneumophila* growth (for recent review, see Berjeaud et al., 2016). However, those compounds, mainly antimicrobial peptides and components of essential oils, don’t originate from the microenvironment of *L. pneumophila*. The survival of *L. pneumophila* in water environments, aside from being sheltered within protists, is also driven by interactions with bacterial inhabitants found as planktonic cells or cells engulfed in mixed community biofilms (Abdel-Nour et al., 2013). *L. pneumophila* can acquire nutrients through synergistic relationships with members of biofilm communities (Tison et al., 1980; Wadowsky and Yee, 1983; Stout et al., 1985; Stewart et al., 2012; Koide et al., 2014; Boamah et al., 2017). Moreover, *L. pneumophila* is capable of surviving by necrotrophic growth on dead cell masses (Temmerman et al., 2006). While interactions promoting *L. pneumophila* survival in oligotrophic water environments are described, competition for nutrients is raging (Abu Khweek and Amer, 2018). Thus, it is reasonable to hypothesize that active molecules are locally produced by biological challengers. Surprisingly, very few papers have described such compounds. In 2008, a study tested 80 aquatic bacterial isolates and showed that 55 displayed antagonistic activity against *L. pneumophila* (Guerrieri et al., 2008). Interestingly, 60 of all tested isolates (75%) were *Pseudomonas* and 43 of them were active against *L. pneumophila* as determined by spot-on-lawn assay. Other species were also described to antagonize the persistence of *L. pneumophila* within biofilms, e.g., *Acidovorax* sp., *Aeromonas hydrophila*, *Burkholderia cepacia*, or *Sphingomonas* sp. (Guerrieri et al., 2008). However, to date, molecules of interest remain uncharacterized. Concomitantly, high-throughput sequencing technologies have enabled a fine characterization of environmental microbiomes such as those dwelling in drinking water distribution systems (Ji et al., 2015; Proctor and Hammes, 2015), shedding light on unexplored and complex microbial communities, potentially hiding new potent antibacterial compounds.

Thus, the aim of the present study was to unravel the potential of those unexplored waterborne bacteria to inhibit the growth of *L. pneumophila*, in order to find new active compounds from natural origin. Environmental aquatic bacteria were thus sampled from five freshwater environments in order to establish a large culturable bacterial collection. Species belonging to various genera and species were then screened for their antagonistic activity toward *L. pneumophila* and identified.

## Materials and Methods

### Water Sampling

Five different water sources were sampled: pond water, swimming pool water, river water, tap water and well water. Each sample was collected in January 2016 in the Vienne department (Nouvelle-Aquitaine, France). Both water samples from a private pond and a private well were collected at Chatellerault (46°49′04″ N, 0°32′46″ E). The river water was sampled from the Vienne River in Bonneuil-Matours (46°40′57″ N, 0°34′17″ E). A sample of an untreated private swimming pool water was collected at Sèvres-Anxaumont (46°34′14″ N, 0°C27′57″ E). Finally, tap water was sampled from the drinking water network of Poitiers (46°34′55″ N, 0°20′10″ E).

### Environmental Bacterial Strains Collection

A volume of 1 mL from each water sample was spread onto R2A agar plates within a maximum of 5 h after the original sampling. All plates were incubated for 72 h at 22, 30, or 37°C. After 72 h, isolated colonies were collected and re-isolated on new R2A agar plates to assure the purity of aquatic isolates. In total, 273 purified isolates were obtained and kept frozen at −80°C until use.

### 16S rRNA Gene Sequencing

Aquatic bacterial isolates were identified by 16S rRNA gene sequencing. For DNA extraction, a colony was suspended in 200 μL of sterile water and the sample was boiled 10 min. Samples were then centrifuged (8000 ×*g*, 15 min) and supernatants containing DNA were kept at −20°C. The 16S rRNA coding gene of bacterial isolates was amplified by PCR with the universal primer set 27F (5′-AGA GTT TGA TCM TGG CTC AG-3′)/786R (5′-CTA CCA GGG TAT CTA ATC-3′), targeting the V1-V4 region of the gene. Amplification was carried out as follows: denaturation at 94°C for 30 s, annealing at 50°C for 30 s, and extension at 72°C for 1 min, for a total of 30 cycles, followed by a final elongation at 72°C for 5 min. Then, PCR products were subjected to 1% agarose gel electrophoresis. For PCR-products clean-up, 1 U/μL of Shrimp Alkaline Phosphatase (M0371S, New England Biolabs) and 10 U/μL of Exonuclease I (M0293S, New England Biolabs) were added to 6 μL of PCR products. DNA sequencing was completed with the ABI Prism BigDye terminator v3.1 sequencing kit (Applied Biosystems, Carlsbad, CA, United States) and then analyzed by an automatic ABI Prism 3730 genetic analyzer (Applied Biosystems, Carlsbad, CA, United States). Sequences were assembled with SerialCloner software and confronted against the nr/nt sequence database using BLASTn (Altschul et al., 1990). All sequences have been deposited in the GenBank database under accession numbers MH591484 to MH591754. Full list of accession numbers is given in Supplementary Table 1.

### Phylogenetic Analysis

Consensus from 16S rRNA gene coding sequences of the 273 isolates were aligned using MUSCLE algorithm (Edgar, 2004a,b). The phylogenetic analysis of 480 bp aligned sequences from the V2–V4 16S gene regions (Position: 201–681) was performed using MEGA V7 software (Kumar et al., 2016). Phylogeny was inferred by maximum likelihood, with 1000 bootstrap iterations to test the robustness of the nodes. The resulting tree was uploaded and formatted using iTOL (Letunic and Bork, 2016).

### Screening for Anti-*Legionella* Activity

*Legionella pneumophila* serogroup 1 Lens CIP 108286 (Cazalet et al., 2008) was used as the reference strain for anti-*Legionella* experiments. All strains were screened for anti-*Legionella* activity by spot-on-lawn assay as described previously (Loiseau et al., 2015). Briefly, *L. pneumophila* was cultured at 37°C in buffered yeast extract (BYE) liquid medium for 72 h under shaking (150 rpm). The pre-culture was then diluted with fresh BYE to a concentration of 10^8^ CFU/mL and a volume of 100 μL was spread onto a buffered charcoal yeast extract (BCYE) agar plate with a cotton swab. Then, 10 μL of suspension of each isolate (obtained from a fresh pre-culture on agar plates) were spotted onto the center of the agar plate. Each plate was then incubated for 48 h at 22, 30, or 37°C. A second incubation step was done at 37°C for 48 h to allow *L. pneumophila* growth. Anti-*Legionella* compounds production was revealed by an inhibition zone around the producer strain. For each tested strain, the diameter of the inhibition area was measured.

### *L. pneumophila* Long-Range Inhibition Assay

This assay was used for environmental bacterial isolates which totally inhibit the growth of *L. pneumophila* on agar plate. A 6-well plate assay was thus designed to physically separate environmental isolates from *L. pneumophila* Lens in order to determine whether emitted volatiles organic compounds (VOCs) could inhibit or not the growth of *L. pneumophila*. Each well was filled with 5 mL of BCYE. Then, 10 μL of a suspension of GFP-expressing *L. pneumophila* Lens (Bigot et al., 2013) (OD_600__nm_ adjusted to 0.1) were spotted onto both upper sides of the plate. Finally, 40 μL of a suspension of selected isolate (OD_600__nm_ adjusted to 1) were spotted onto the upper center of the plate. Depending of the tested isolate, plates were incubated for 48 h at 22, 30, or 37°C. A second step of incubation was done for 48 h at 37°C. Anti-*Legionella* activity mediated by VOCs production was quantified by measuring the fluorescence of GFP with a TriStar^2^ LB 942 Microplate Reader (Berthold Technologies, Bad Wildbad, Germany).

### Data Analysis

For analyzing the distribution of diameters among the five water sub-collections, violin plots were constructed using R environment with the package “ggplot2” (Wickham, 2009). To assess the alpha-diversity within each water collection, the Shannon diversity index (H) was calculated based on relative abundance for assigned genera. To evaluate the evenness of each community, Pielou’s index (J) was computed. Both indexes were calculated in R environment using the package “Vegan” (Oksanen et al., 2018). Finally, a heatmap representing inhibition diameters distribution among bacterial genera was constructed using Plotly online software^1^.

## Results

### Waterborne Bacteria Exhibit a High Proportion of Anti-*Legionella* Compounds Producers

**FIGURE 1:**
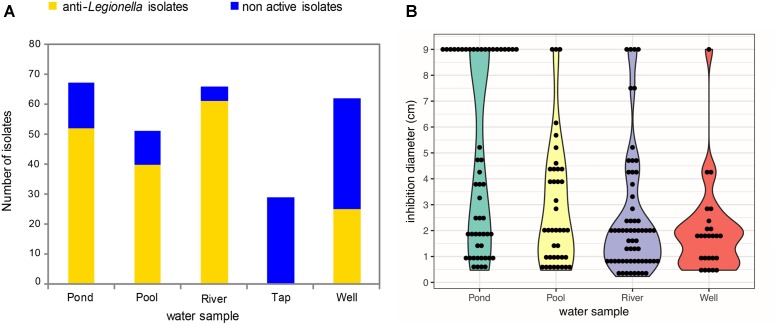
Abundance and distribution of active anti-*Legionella* bacterial isolates and their inhibition diameters among the five water sub-collections. **(A)** Active isolates are colored in yellow while non-active isolates are colored in blue. The screening was realized by using spot and lawn assay method. **(B)** Violin Plot distribution of inhibition diameters among anti-*Legionella* isolates. Since there is no active isolate within the tap water, the corresponding sub-collection was not represented.

Firstly, a total of 273 bacterial isolates were recovered and purified from the five different freshwater sources (Pond, Pool, River, Tap, and Well) used in this study. For each water sample, between 51 and 67 isolates were obtained per mL of a given water sample except for the tap water sample for which the cultivable bacterial biomass was lower (29 strains) (Figure 1A). Secondly, the collection of aquatic bacterial isolates was screened for the ability to produce anti-*Legionella* compounds using an agar plate assay (repeated at least three times per isolate). Among the 273 isolates, 178 (65.2%) were shown to be active against *L. pneumophila* Lens CIP 108286 (Figure 1A). Furthermore, those active isolates were not equally distributed among the five water sub-collections: 52/67 from the pond sub-collection (77.7% of isolates), 40/51 from the pool sub-collection (78.4% of isolates), 61/66 from the river sub-collection (92.4% of isolates), 25/62 from the well sub-collection (39.7% of isolates) and 0/29 for the tap sub-collection (Figure 1A). Interestingly, various inhibition diameters were observed among active isolates, ranging from 0.4 to 9 cm (Figure 1B). However, for each water sub-collection, most of inhibition diameters values were comprised between 0.4 and 2 cm. This inhibition profile was mainly observed for the well water and the river water sub-collections representing 68 and 60.6% of the total active isolates, respectively. Another point of interest is the full inhibition profile (diameter of 9 cm) displayed by several isolates from the collection (Figure 1B). Surprisingly, those isolates were not evenly distributed but were mostly found in the pond water sub-collection (38.4% of total active isolates). In all other water sub-collections, the proportion of isolates displaying a total inhibition profile was lower (7.5, 6.6, and 4% of total active isolates in the pool, the river and the well water sub-collections respectively).

### Gammaproteobacteria and Firmicutes Are the Major Clades of Anti-*Legionella* Bacteria

**FIGURE 2:**
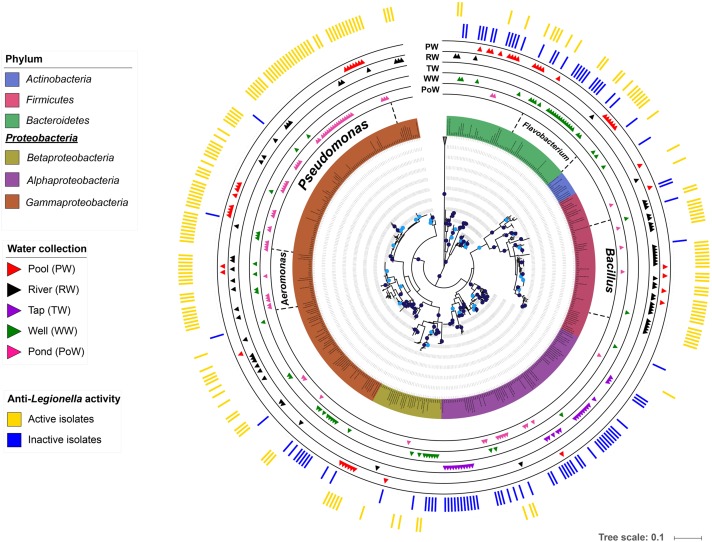
Phylogenetic tree of anti-*Legionella*-associated environmental bacteria. The phylogenetic analysis was performed using the maximum likelihood phylogenetic method on MEGA v7 and the tree was viewed with iTOL (Letunic and Bork, 2016). Branches with bootstrap values (1000 replicates) higher than 70% are marked with dark blue dots, and those between 60 and 70% with light blue dots (The tree did not have branches with bootstrap value less than 60%). Environmental bacterial strains are color-coded by phyla, except for the Alphaproteobacteria, Betaproteobacteria, and Gammaproteobacteria which are depicted at the class level. The outer colored bands show the anti-*Legionella* profile with active isolates in yellow and inactive isolates in blue. The five internal bands show the distribution of each isolate among the five environmental water sub-collections (pond, pool, river, tap, and well).

In order to explore taxonomic affiliations of bacterial isolates, a systematic sequencing of the 16S rRNA coding gene sequence was performed. Resulting sequences were used to reconstruct a phylogenetic tree presented in Figure 2. All bacterial isolates were classified within 4 phyla, namely Proteobacteria (179/273), Bacteroidetes (48/273), Firmicutes (43/273), and Actinobacteria (3/273). In regards to the anti-*Legionella* activity, the Firmicutes phylum and the Gammaproteobacteria class stood out as clades containing the highest proportion of active isolates, as 89.8% of Gammaproteobacteria and 88.4% of Firmicutes showed inhibition of *Legionella* growth. Conversely, the Alphaproteobacteria class, for which most isolates originated from the tap water sub-collection, harbored the lowest proportion of active isolates (9.1%) (Figure 2). Genera such as *Bradyrhizobium* and *Novosphingobium*, which were exclusively isolated from the tap water, did not show any anti-*Legionella* activity. Overall, members of the Gammaproteobacteria class were the most frequently isolated bacteria, regardless of the environmental water source. Within this class, *Aeromonas* and *Pseudomonas* were the most represented genera, which were found in all samples except in the tap water sub-collection. Together with *Flavobacterium* (Bacteroidetes) and *Bacillus* (Firmicutes), these four genera dominated the collected bacterial community as they represented 56.4% (154/273) of all isolates of the collection (Figure 2). Within those 154 isolates, 89% (137/154) were found active thus representing 100% of *Aeromonas* isolates, 94.7% of *Bacillus* isolates, 51.8% of *Flavobacterium* isolates and 97.1% of *Pseudomonas* isolates (Figure 2).

### The Well Water Sub-collection Harbors the Most Diverse Cultivable Bacterial Flora

**FIGURE 3:**
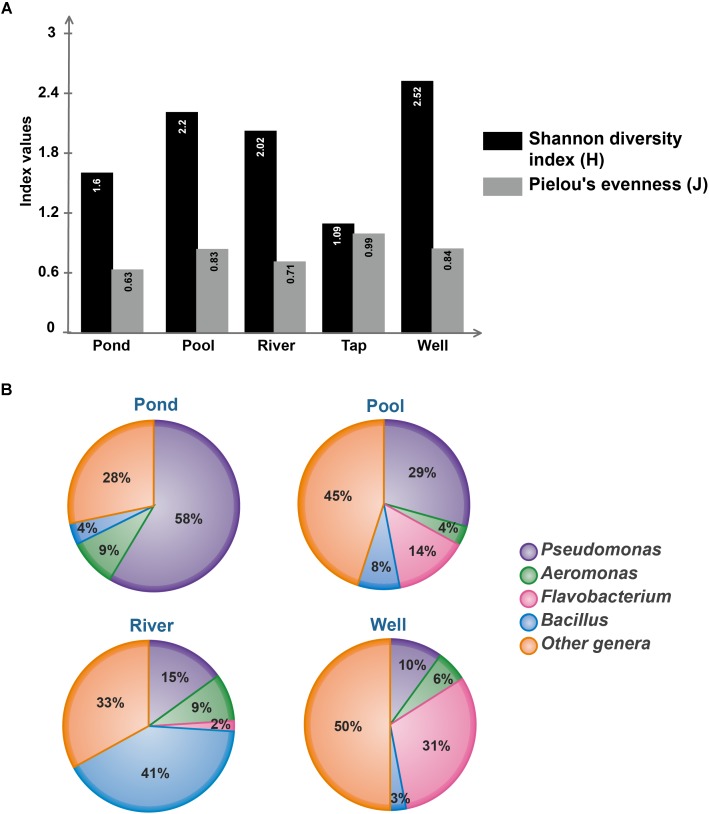
Microbial diversity of environmental water sub-collections. **(A)** Bacterial communities’ richness and evenness from each water sub-collection, as estimated by Shannon and Pielou indexes, respectively. Calculation were performed using R package vegan (Oksanen et al., 2018). **(B)** Relative abundances of the most represented (i.e., dominant) genera in each water sub-collection. The tap water condition is not represented by a circular diagram since this sub-collection harbors only three bacterial genera, namely *Bradyrhizobium*, *Sphingobium*, and *Sphingomonas.*

With the aim to estimate the bacterial diversity and species evenness within water sub-collections, Shannon H diversity and Pielou’s evenness indexes were calculated (Figure 3A). Concerning the bacterial richness, the well water contains the most diverse community (*H* = 2.52; 12 equally common species) while the tap water is the lowest (*H* = 1.09) which is not surprising because this sub-collection contains only three genera. The pool water and the river water richness were similar (*H* ranging from 2.02 to 2.2) whereas the pond water is poorly diverse with a *H* index = 1.6 (Figure 3A). Furthermore, the less even community identified corresponds to the pond water (*J* = 0.63), suggesting the dominance of few genera, in addition to a more distinct bacterial community assemblage when compared to others water samples. In the tap water, no dominant species could be identified as the community was very even (*J* = 0.99), which is likely to be linked with the low richness of this sub-collection (Figure 3A). The well water and the pool water showed similar J index (0.84 and 0.83, respectively), suggesting the absence of highly dominant species based on the presented dataset (Supplementary Table 1). Those pieces of data indicate that both communities differ in bacterial diversity but do not contain much specific genera. To complete this analysis and further identify potential dominant taxa, relative abundances of the four main bacterial genera (*Aeromonas, Bacillus*, *Flavobacterium*, and *Pseudomonas*) present in the water sub-collections were determined (Figure 3B). Altogether, those genera represented up to 70% of the total bacterial community. Both their distribution and abundance show a high amount of *Pseudomonas* isolates in the pond water sub-collection (58% of all isolates), *Bacillus* isolates in the river water sub-collection (41% of all isolates) and *Flavobacterium* isolates in the well water sub-collection (31% of all isolates) (Figure 3B). *Aeromonas* isolates were present in all sub-collections, although less abundantly than the three others genera.

### Diffusible Molecules and/or Volatile Compounds Are the Cause of the Anti-*Legionella* Activity

**FIGURE 4:**
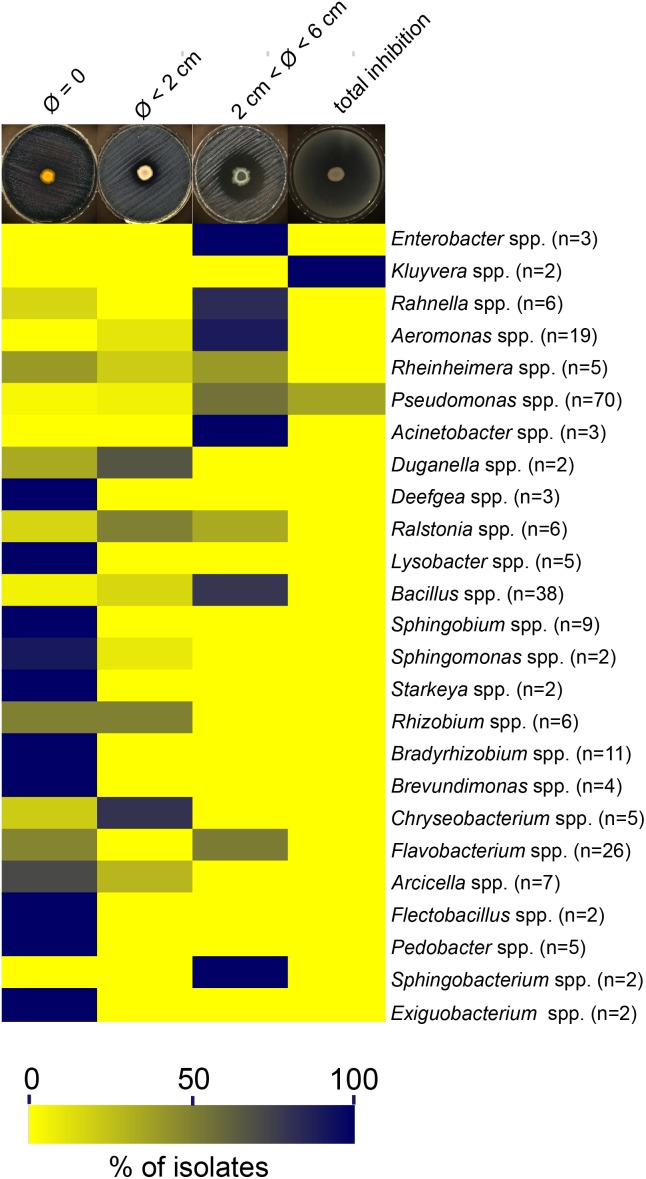
Distribution of inhibition diameters among bacterial genera. The heatmap was constructed using relative abundance of each inhibition profile among all genera. The letter “n” corresponds to the total number of bacterial isolates for a given genus. Genera represented by only one isolate are not included in this heatmap and are detailed in Table 1.

To decipher the underlying variety of anti-*Legionella* activity displayed by active strains, a heatmap was drawn, highlighting clusters according to the diameter of inhibition. Overall three clusters were identified, showing diameters of inhibition (i) inferior to 2 cm, (ii) between 2 and 6 cm, and (iii) equal to 9 cm (total inhibition), in addition to strains devoid of anti-*Legionella* activity (Figure 4 and Table 1). Among 25 active genera, 17 showed a specific inhibition profile with a constant diameter of inhibition (i.e., induced by more than 70% of all strains within the genus). A total absence of inhibition could be consistently observed for *Flectobacter*, *Pedobacter* (Bacteroidetes), *Exiguobacterium* (Firmicutes), *Sphingobium*, *Starkeya*, *Bradyrhizobium, Brevundimonas* (Alphaproteobacteria), *Deefgea* (Betaproteobacteria) and *Lysobacter* (Gammaproteobacteria). Profiles corresponding to diameters of inhibition inferior to 2 cm were observed for *Arthrobacter* (Actinobacteria), *Chryseobacterium* (Bacteroidetes), *Brevibacillus* (Firmicutes), *Achromobacter*, *Acidovorax*, *Duganella*, and *Rhodoferax* (Betaproteobacteria). Profiles resulting in diameter of inhibition between 2 and 6 cm were observed for a large variety of Gammaproteobacteria isolates, affiliated to *Acinetobacter*, *Aeromonas*, *Enterobacter*, *Escherichia*, *Erwinia*, *Klebsiella*, and *Rahnella* genera; other clades were less diversely represented with *Lysinibacter* (Actinobacteria), *Sphingobacterium* (Bacteroidetes) and *Bacillus* (Firmicutes). Other genera could not be classified in such clusters, as large intra genus variability could be observed (e.g., *Rheinheimera*, *Ralstonia*) (Figure 4).

**Table 1:** Inhibition diameters for genera with only one representing isolate in the collection.

Genus	Diameter (cm)
*Azospirillum*	∅ = 0
*Curtobacterium*	∅ = 0
*Novosphingobium*	∅ = 0
*Pseudacidovorax*	∅ = 0
*Pseudoxanthomonas*	∅ = 0
*Rhodococcus*	∅ = 0
*Shewanella*	∅ = 0
*Staphylococcus*	∅ = 0
*Achromobacter*	∅ < 2
*Acidovorax*	∅ < 2
*Arthrobacter*	∅ < 2
*Brevibacillus*	∅ < 2
*Rhodoferax*	∅ < 2
*Escherichia*	2 < ∅ < 6
*Erwinia*	2 < ∅ < 6
*Klebsiella*	2 < ∅ < 6
*Lysinibacillus*	2 < ∅ < 6

**FIGURE 5:**
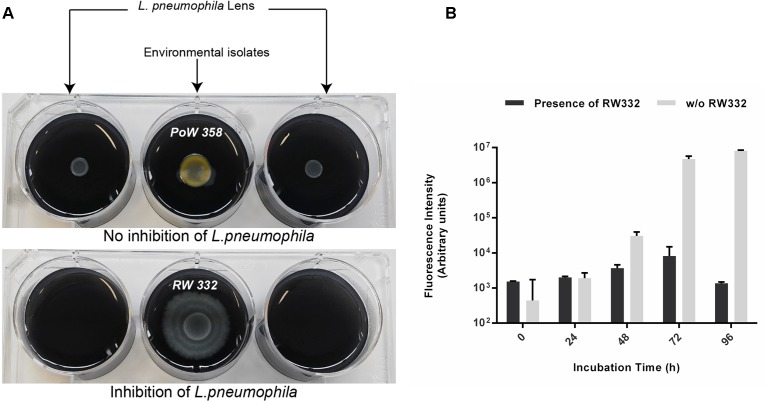
Long distance way inhibition of GFP-expressing *L. pneumophila* through the production of volatile compounds produced by several *Pseudomonas* isolates. *L. pneumophila* was spread on both upper sides of the 6 wells plate. Each isolate was spread on the center of 6-well plates. **(A)** On the left microtiter plate, *Pseudomonas* PoW358 does not exhibit any anti-*Legionella* activity while on the right microtiter plate, *Pseudomonas* RW332 exhibits a volatile compounds-mediated growth inhibition. **(B)** Fluorescence quantification emitted by GFP-expressing *L. pneumophila* Lens in presence and absence of the strain RW332 (*n* = 3).

Unexpectedly, isolates belonging from two genera were found to totally inhibit the growth of *L. pneumophila* since no colony was detected on entire Petri-dishes when compared with *L. pneumophila* grown alone: *Kluyvera* and *Pseudomonas* (Figure 4). Among the 70 isolates of *Pseudomonas*, only 26 (37.1%) were concerned while the two isolates of *Kluyvera* (100%) available in the collection exhibited this capacity. Those results suggest that at least one highly diffusible and/or a volatile antagonistic molecule could be produced. To further investigate this possibility, we designed a 6-well antagonistic assay enabling the quantification of long-range aerial interference between *Kluyvera*/*Pseudomonas* and physically separated GFP-expressing *L. pneumophila* Lens. Thus, the volatile activity could be visually estimated as a function of *L. pneumophila* growth (Figure 5A). The use of a GFP-expressing *L. pneumophila* strain also allowed for a quantitative estimate of growth inhibition through fluorescence intensity measurements. This experimental design allowed us to verify simultaneously both hypotheses and an example is given in Figure 5B for the strain RW332. While no *Kluyvera* isolates were shown to produce volatile compounds capable of inhibiting *L. pneumophila* growth, 6 out of 26 *Pseudomonas* isolates displayed this capacity and may produce at least one anti-*L. pneumophila* volatile compound. In contrast, the 20 others *Pseudomonas* as well as the 2 *Kluyvera* isolates may have produced one or more highly active diffusible antagonistic molecules.

## Discussion

Artificial water systems provide suitable conditions for growth and multiplication of waterborne pathogens including *L. pneumophila*, the causative agent of LD (Van Heijnsbergen et al., 2015). Characteristics shared by some waterborne pathogens include proliferation in biofilms, uptake, survival, and proliferation within an amoeba host and disinfectant resistance (e.g., chlorine, chloramine). The survey and the control of disinfection by-products are key factors for minimizing people exposure while maintaining satisfactory disinfection processes. However, the array of investigated compounds displaying anti-*Legionella* activity remains limited (Berjeaud et al., 2016). When available, those compounds were purified from organisms not found in the microenvironment of *L. pneumophila*. Thus, in this study, we established a vast freshwater bacterial collection in order to investigate the presence of *L. pneumophila-*inhibiting bacterial strains sharing the same ecological niches.

Five freshwater sources were sampled (bulk water) to increase the biodiversity of harvested bacteria and potentially active isolates. Among the 273 isolates, 178 (65.2%) were shown to be active against *L. pneumophila* as revealed by the presence of an inhibition area on Petri dishes. The diversity of inhibition diameters obtained could be linked to many factors such as the physical and chemical properties of active compounds, the physiological state of the target, the metabolic activity of the producing strain or the number of compounds that are produced. Nevertheless, those pieces of data are in agreement with those obtained by Guerrieri and coworkers who isolated 51 strains (51/75; 68%) active against *L. pneumophila* from different tap water samples (Guerrieri et al., 2008). It is, however, important to stress that 75% of tested strains in the above-mentioned study belonged to the *Pseudomonas* genus and the biodiversity of their collection was somewhat restrained. Nonetheless, they showed that among their 20 non-*Pseudomonas* remaining strains (9 *Acinetobacter* spp., 2 *A. faecalis odorans*, 2 *A. hydrophila*, 2 *B. cepacia*, and 1 *Flavobacterium* spp.), 12 (60%) were active against *L. pneumophila*. In our study 70 strains (25.6%) belong to the *Pseudomonas* genus and, as expected, all but 2 displayed antagonistic activity. Among active genera in the collection, 20 are newly described as anti-*Legionella* to the best of our knowledge. Only *Aeromonas* (Cotuk and Dogruoz, 2005; Guerrieri et al., 2008)*, Bacillus* (Loiseau et al., 2015), *Acinetobacter*, *Flavobacterium*, and *Pseudomonas* (Guerrieri et al., 2008) have been previously reported. Moreover, for many of them, such as *Chryseobacterium*, *Ralstonia*, or *Rheinheimera*, we have collected more than one active isolate, hence reinforcing the conservation of such antagonistic aspect across isolates from the same genus. However, it has to be noted that because R2A was used to recovered water bacteria, other genera that cannot grow on this medium and that might produce anti-*Legionella* compounds could not be detected in this study.

The diversity of bacteria isolated in this study was shown to be modulated by the water source that was sampled. The well water corresponded to the most diverse source, whereas the tap water showed the lowest diversity. Apart from the pond water where *Pseudomonas* spp. accounted for more than half of the isolates, no highly dominant taxa were found in other samples. Indeed, the source-specific diversity contributed to gather a large and diversified collection of bacterial isolates, affiliated to four phyla, namely Actinobacteria, Bacteroidetes, Firmicutes and Proteobacteria. Most active isolates, 153/178 (86%) were recovered from pond, pool and river water samples accounting for 77.4–92.4% of all strains. Surprisingly, no active strain was isolated from the tap water sample, which is also the less diverse bacterial sub-collection with only three genera (*Bradyrhizobium*, *Sphingobium*, and *Sphingomonas*) maybe because it is the only one sample coming from a treated water system. This result may suggest that water treatment remove anti-*Legionella* bacterial species from the bulk water. However, further investigations are required to elucidate whether such treatments could indirectly favor *Legionella* sp. persistence by eliminating natural competitors. The three most represented genera, i.e., *Bacillus*, *Pseudomonas*, and *Flavobacterium*, all displayed anti-*Legionella* activity, at least for 50% of isolates. As suggested by the sequence comparisons and phylogeny, isolates affiliated to these three genera are highly likely to represent a various set of distinct species and strains. However, because of the underlying limitations of 16S rRNA-based bacterial identification, this diversity is surely underestimated. Other methods such as multi-locus sequence typing or whole genome sequencing would indubitably give more information in this regard.

The literature concerning the chemical nature of diffusible molecules responsible for anti-*Legionella* activity still remains scarce to this day. Recently, surfactin, the most known and well-studied biosurfactant from *Bacillus subtilis*, has been shown to exhibit a potent activity toward many *Legionella* species including the Lens strain (CIP 108286) (Loiseau et al., 2015). Moreover, *Legionella* bacteria appeared highly sensitive to this lipopeptide (MICs of 1–4 μg/mL) in comparison with the few susceptible species reported so far (Berjeaud et al., 2016). Biosurfactants, which are well-studied microbial metabolites, are often classified according to their chemical composition and their molecular weight. These low (i.e., glycolipids, phospholipids, and lipopeptides) and high-molecular weight (i.e., polysaccharides, proteins, lipoproteins, and LPS) compounds are produced by many bacterial genera including *Acinetobacter*, *Bacillus* and *Pseudomonas* (Płociniczak et al., 2011). As *L. pneumophila* was previously described to be sensitive to various membrane-active biomolecules (Berjeaud et al., 2016), lipopeptides from *Bacillus* and rhamnolipids from *Pseudomonas* might be potent anti-*Legionella* weapons. Additionally, many antimicrobial peptides (AMPs) were found capable of killing *Legionella* sp. (Berjeaud et al., 2016). Those AMPs were characterized from various organisms like warnericin RK and PSM from Staphylococci (Marchand et al., 2011; Verdon et al., 2008), Ci-PAP-A22 and Ci-MAM-A24 from marine *Ciona intestinalis* (Schlusselhuber et al., 2015), Armadillidin H from the woodlouse *Armadillidium vulgare* (Verdon et al., 2016) and a defensin from the greater wax moth *Galleria mellonella* (Palusińska-Szysz et al., 2012). However, very few bacterial genera identified in our study were described to produce AMPs. To our knowledge, only *Aeromonas* (Tasiemski et al., 2015), *Escherichia* (Rebuffat, 2012), and *Klebsiella* (de Lorenzo and Pugsley, 1985) were reported so far. As those AMPs have never been tested against *L. pneumophila*, we hypothesize that they might eventually be involved in the antagonism ability of such isolates. However, further biochemical investigations would be necessary to purify and characterize such AMPs and to explore their potential use as biocontrol agents against *L. pneumophila*. Ultimately, many biomolecules are active against *L. pneumophila*, including molecules that are described to exhibit a poor antibacterial potency (e.g., warnericin RK or surfactin) (Berjeaud et al., 2016). *L. pneumophila* might have some particularities that could explain this sensitivity and part of the answer definitely lies in the composition of its envelope. For example, this bacterium contains an elevated amount of branched chain fatty acids and this characteristic was linked to its sensitivity to an antimicrobial peptide (Verdon et al., 2011). In the same way, detergents are very effective as anti-*Legionella* agents (Verdon et al., 2009) highlighting the crucial role played by some cell surface components. As underlined by those studies, the sensitivity *L. pneumophila* to various biomolecules is poorly understood because there is a current lack of mechanistic data (Berjeaud et al., 2016).

The well antagonistic assay used to physically separate *Kluyvera*/*Pseudomonas* isolates from GFP-expressing *L. pneumophila* Lens, allowed us to detect the production of at least one anti-*Legionella* volatile organic compound (VOC) from 6 *Pseudomonas* strains since aerial exposure to volatile molecule(s) released from those strains inhibited the growth of *L. pneumophila*. It is the first report published so far of such a long-range aerial interference between *Pseudomonas* and *Legionella*. VOCs and volatilomes have been studied primarily in a context of inter-kingdom responses and their roles in bacterium-to-bacterium interactions are largely unexplored (Schulz and Dickschat, 2007). However, several studies suggested that bacterial VOCs could influence some phenotypes such as colony morphogenesis, biofilm formation, pigment production or antibiotic resistance (Bernier et al., 2011; Létoffé et al., 2014). The reasons why microorganisms produce volatiles remain unclear even if numerous functions such as communication, quorum sensing mechanisms and defense have been suggested (Kai et al., 2009). Nevertheless, these assumptions are difficult to demonstrate. Indeed, some bacterial volatiles have antifungal activity (Popova et al., 2014; Lo Cantore et al., 2015) while others can also serve as signals, attracting or repelling different organisms. Furthermore, they can also induce resistance mechanism toward bacterial pathogens in plants or promote their growth (Farag et al., 2013). To date, none of these molecules was described as being anti-*Legionella*. Further investigations are needed to extract and analyze these molecules in order to decipher their mechanism of action. However, the role played by those volatiles compounds in an aquatic environment remains completely unknown.

In summary, the high proportion of anti-*Legionella* isolates recovered emphasizes that freshwater environments are not favorable to *L. pneumophila* in contrast to treated manmade water settings and highlights the key role played by biofilms and protists that shelter this human pathogen from competitors and harsh conditions (e.g., low temperature, presence of biocides). Thus, by collecting a vast array of *L. pneumophila* competitors, this study represents a first step and a prerequisite for further identification of potentially novel and active biomolecules.

## Author Contributions

M-HC, JM-B, and JV conceived and designed the study. M-HC performed experiments with the help of AL for antagonism experiments. M-HC and VD analyzed the data. J-MB and JV supervised the work. All authors edited the final manuscript.

## Conflict of Interest Statement

The authors declare that the research was conducted in the absence of any commercial or financial relationships that could be construed as a potential conflict of interest.

## Footnotes

1 https://plot.ly/
