# Supplementary material for: Exploiting the Richness of Environmental Waterborne Bacterial Species to Find Natural Legionella pneumophila Competitors
Source: Front Microbiol. 2019 Jan 15;9:3360. doi: 10.3389/fmicb.2018.03360 (PMC6340971; doi:10.3389/fmicb.2018.03360)
Supplement: Supplementary file 1 [file Table_1.DOC]

**Supplementary Table 1** Full list of 16S rRNA coding sequences accession numbers
